# Harnessing Oxidizing Potential of Nickel for Sustainable Hydrocarbon Functionalization

**DOI:** 10.3390/molecules29215188

**Published:** 2024-11-02

**Authors:** Thomas M. Khazanov, Anusree Mukherjee

**Affiliations:** 1 Department of Chemistry and Chemical Biology, Cornell University, Ithaca, NY 14853, USA; tmj49@cornell.edu; 2 Department of Chemistry and Geosciences, Jacksonville State University, 700 Pelham Rd N, Jacksonville, AL 36265, USA

**Keywords:** hydrocarbon functionalization, oxygen activation mediated by Ni, catalysis by Ni complexes

## Abstract

While the oxidative chemistry of transition metals such as iron and copper is a highly developed area of investigation, the study of similar chemistry with nickel is much younger. However, nickel offers rich coordination chemistry with oxygen and other oxidants and is a promising avenue of research for applications such as sustainable hydrocarbon functionalization. Herein, we summarize the progress made recently in nickel coordination chemistry relevant to hydrocarbon functionalization and offer our perspectives on open questions in the field.

## 1. Introduction

Over the last decade, the global energy supply has undergone significant diversification, such that the reserve of natural gas has greatly increased owing to improved methods of recovery and discovery [1,2]. Because gases are more difficult to handle and transport, the majority of natural gas is flared during the oil extraction process, representing an immense waste of energy on the global scale. Gas-to-liquid (GTL) conversion of methane, the major component of natural gas, is conducted only on a limited scale and the development of new GTL methods that are more efficient and economical remains an active area of research. While most methane is currently obtained from fossil sources, renewable sources of methane are under development, namely in the forms of biomethane and power-to-gas technology [3]. Similarly, most of the global chemical feedstock comes from the refinement of petrochemicals, and thus renewable sources of light alkane precursors are necessary.

However, the use of methane as fuel still results in the formation of carbon dioxide. In the long term, any sustainable solution to the fossil fuel problem must also consider carbon dioxide, itself a potentially valuable light carbon precursor. Therefore, it is necessary to develop sustainable methods for both the oxidation of carbon in its lowest oxidation state (as in methane) as well as the reduction of carbon in its highest oxidation state (as in carbon dioxide), the former being the focus of this review.

Transition metal complexes have been widely investigated as catalysts for hydrocarbon activation and functionalization. The study of enzymes such as cytochrome P450 and methane monooxygenase have inspired a rich literature concerned with hydrocarbon functionalization in the case of iron and copper. Nickel, on the other hand, remains considerably less explored in the context of hydrocarbon functionalization, though other group 10 metals such as platinum and palladium have long been employed for hydrocarbon functionalization, including for methane oxidation [4,5]. In this article, we have chosen to highlight nickel-mediated hydrocarbon functionalization in homogeneous systems and its potential for sustainable oxidation chemistry.

The role of nickel in hydrocarbon functionalization in this article is presented through three lenses: hydrocarbon functionalization under catalytic conditions, fundamental reactivity studies under stoichiometric conditions, and oxidative chemistry carried out by nickel-containing metalloenzymes. With this in mind, we have limited the scope of this minireview primarily to the progress made in the field with mononuclear nickel precursors over the last two decades.

## 2. Catalytic Hydrocarbon Functionalization

Since the early 2000’s, a quite comprehensive body of literature has developed regarding the development of catalysts for the selective oxidation of alkanes under mild conditions. Though previously much attention had been paid to heme and non-heme iron complexes owing to their high catalytic efficiency and biological relevance, it was shown that across a series of manganese, iron, cobalt, and nickel complexes supported by the tetradentate ligand tris(2-pyridylmethyl)amine (TPA, **L^1^**), the Ni^II^(TPA) complex was the most efficient oxidation catalyst [6]. This complex oxidized cyclohexane, cyclooctane, adamantane, and ethyl benzene with high selectivity using *m*-chloroperbenzoic acid (*m*-CPBA) as the oxidant precursor [6]. Alcohols were obtained as oxidation products with high turnover number (TON) and an alcohol/ketone (A/K) ratio of 5 or greater was obtained for all substrates investigated. In the case of adamantane, high regioselectivity between the tertiary (3°) carbon and secondary (2°) carbon was observed, with a 3°/2° ratio of 12.5 [6]. Similarly, the benzylic carbon in ethylbenzene was selectively oxidized [6]. Based on the existing literature on the gas phase reactivity of transition metal oxide cations (MO^+^) and the high A/K ratio observed, it was concluded that an [NiO]^+^ intermediate was the most likely oxidant [6,7].

**Figure 1:**
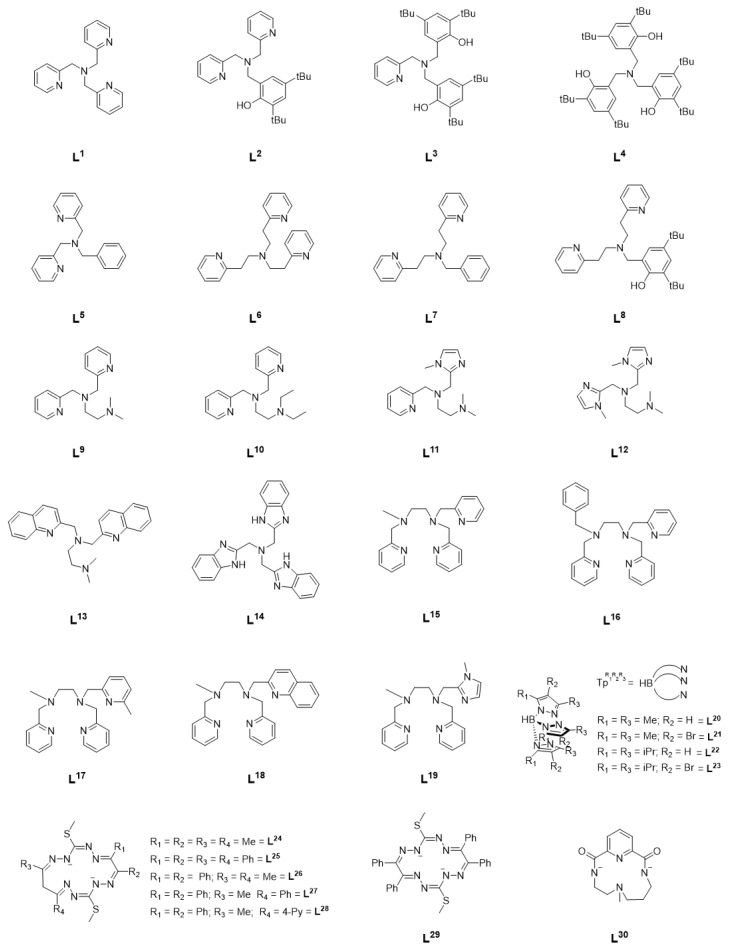
Ligands supporting Ni^II^ complexes in catalytic hydrocarbon functionalization studies.

In a series of follow-up studies by Itoh et al., the effect of ligand electronics on catalytic activity was systematically investigated by varying the denticity, linker length, and donor atoms of the TPA ligand (Figure 1, **L^2^**–**L^8^**) [8]. Substitution of one of the pyridine rings with a functionalized phenol, as in **L^2^**–**L^4^** for example, led to mixed results. In the case of **L^2^**, an increase in alcohol TON was observed but substitution of all the TPA pyridines resulted in a decrease in TON but an A/K ratio of 65, higher than any other complex in this series (Table 1: entries 4, 5, and 7). When the ligand denticity was reduced from tetradentate (**L^1^**) to tridentate (**L^5^**), a decrease in efficiency was observed, though there was little change in A/K ratio (Table 1: entries 1, 2, and 8). Variation of the linker length did little to improve catalytic efficiency, though it did lead to an increase in A/K ratio even in the case of **L^7^**, a tridentate ligand, and **L^8^**, in which one of the pyridines has been replaced with a substituted phenol (Table 1: entries 1, 2, 9–13). Shorter methylene linkers overall resulted in higher catalytic efficiency, while longer ethylene linkers resulted in higher selectivity. In a series of copper complexes supported by the same ligands, it was demonstrated that the methylene linkers resulted in greater electron-donating ability of the coordinating pyridine than with ethylene linkers, and that this may be a possible reason for the difference in reactivity [6,9]. In each case, higher catalytic efficiency was observed when acetate was included as the counter anion. This was potentially attributed to the lower basicity of NO_3_^−^ relative to AcO^−^, resulting in slower ligand exchange of NO_3_^−^ with *m*-CPBA [8]. The high selectivity observed for each complex suggest a metal-based oxidant such as a [NiO]^+^ species, rather than free radicals, though the reactive intermediate could not be isolated and characterized [6,8,10].

Based on the observations of the Itoh group, Palaniandavar et al. undertook a systematic investigation of alkane oxidation with *m*-CPBA using nickel complexes supported by two families of ligands (**L^9^**–**L^14^** and **L^15^**–**L^19^**) [11,12]. In the series of tetradentate ligands **L^9^**–**L^14^**, the pyridines of TPA were replaced with amines (**L^9^**–**L^10^**), benzimidazoles (**L^11^**–**L^12^**, **L^14^**), or quinolines (**L^13^**). All six complexes were able to oxidize cyclohexane, adamantane, cumene, and ethylbenzene with high TON and modest A/K ratio. In the case of adamantane, similar regioselective oxidation of 3° carbons over 2° was observed as with Ni^II^(TPA). Under similar reaction conditions (Table 1: entries 3 and 15), a modest increase in catalytic efficiency was observed with **L^9^** compared to **L^1^**. Replacing the amine functionality with benzimidazole or quinoline resulted in a reduction in catalytic efficiency (Table 1: entries 15–20), and, taken together, it was concluded that coordinating amines results in an increase in Lewis acidity of the nickel center, thus encouraging the binding of *m*-CPBA and resulting in higher catalytic activity, while replacement of the pyridine moieties with benzimidazolyl moieties leads to an increase in electron density on the nickel center and disfavors binding of *m*-CPBA relative to **L^1^**. Furthermore, the inclusion of large quinolyl/benzimidazolyl moieties may inhibit binding of *m*-CPBA through steric interactions as well. In all cases, inclusion of a fifth coordinating atom resulin a decrease in efficiency relative to **L^9^** under similar reaction conditions (Table 1: entries 15, 21–25). Both studies concluded that the active oxidant was an [NiO]^+^ species, and a computational investigation of the pentadentate complexes described the electronic structure of such an intermediate as a high-spin Ni^II^-oxyl species with a low-lying *S* = ½ excited state [11,12].

Further investigation from Hikichi et al. expanded the studies above to include a series of substituted trispyrazolylborate ligands (**L^20^**–**L^23^**) [13]. Dinuclear precursors supported by these ligands were treated with *m*-CPBA to yield an oxidant that was capable of oxidizing cyclohexane in the case of ligands **L^20^** and **L^21^**, with a larger A/K ratio being observed in the case of **L^21^** (Table 1: entry 27). No oxidation of cyclohexane was observed with ligands **L^22^** and **L^23^**, which was attributed to the steric bulk of the ligand. It is proposed that although the starting complexes were dinuclear in nature, introduction of *m*-CPBA results in a mononuclear Ni^II^-acylperoxo intermediate that ultimately forms an [NiO]^+^ species as the primary oxidant, though the Ni^II^-acylperoxo species may also be competent for cyclohexane oxidation, as recently reported [14]. Further work from Hikichi et al. also demonstrated catalytic hydrocarbon oxidation using bis(oxazoline)-supported nickel complexes [15].

More recently, a series of Ni^II^ complexes supported by a family of octaazamacrocyclic ligands (**L^24^**–**L^29^**) were investigated for C-H bond activation, though under very different conditions [16]. Microwave-assisted, solvent-free oxidation of cyclohexane was achieved with *tert*-butyl hydroperoxide (TBHP) as the oxidant. The reactivity could be neatly correlated with their measured reduction potentials, with the highest reactivity observed in the case of the complex with the highest reduction potential. In this study, the ligands employed showed redox noninnocent behavior and the oxidation was postulated to proceed through a ligand-centric mechanism that resulted in the formation of free radicals rather than formation of a metal-centered oxidant. When supported by tetradentate macrocyclic ligand **L^30^**, however, addition of *m*-CPBA did result in a metal-centered oxidant that the authors formulate as a formally Ni^III^-oxyl species [17]. Interestingly, upon introduction of NaOCl/AcOH to the same complex, catalytic chlorination could be observed [18].

**Table 1:** Oxygenation of cyclohexane with *meta*-chloroperbenzoic acid catalyzed by Ni^II^ complexes.

Entry	Compound	Alcohol TON	Ketone TON	A/K	Reaction Time (h)	Reference
1	^a^ [Ni^II^(L^1^)(OAc)(H_2_O)]BPh_4_	587	69	8.5	1	[8]
2	^a^ [Ni^II^_2_(L^1^)_2_(μ-NO_3_)_2_](BPh_4_)_2_	544	68	8.0	1	[8]
3	^b^ [Ni^II^(L^1^)(CH_3_CN)_2_](BPh_4_)_2_	450	55	8.1	2	[11]
4	^a^ [Ni^II^(L^2^)(OAc)(MeOH)]BPh_4_	657	88	7.5	1	[8]
5	^a^ [Ni^II^(L^2^)(NO_3_)(MeCN)]NO_3_	567	61	9.3	1	[8]
6	^a^ [Ni^II^(L^3^)(OAc)_2_]	759	99	7.7	2	[19]
7	^a^ [Ni^II^(L^4^)(TMG)]	295 *	5 *	65.0	2	[19]
8	^a^ [Ni^II^(L^5^)(OAc)_2_(H_2_O)]	455	69	6.6	1	[8]
9	^a^ [Ni^II^(L^6^)(OAc)]BPh_4_	257	8	32.1	1	[8]
10	^a^ [Ni^II^(L^6^)(NO_3_)]BPh_4_	46	1	46	1	[8]
11	^a^ [Ni^II^(L^7^)(OAc)(H_2_O)]BPh_4_	265	7	37.9	1	[8]
12	^a^ [Ni^II^(L^8^)(OAc)]	332	8	41.5	1	[8]
13	^a^ [Ni^II^(L^8^)(NO_3_)]	182	4	45.5	1	[8]
14	^b^ [Ni^II^(L^9^)(H_2_O)(CH_3_CN)](ClO_4_)_2_	479	42	4.6	2	[11]
15	^b^ [Ni^II^(L^9^)(CH_3_CN)_2_](BPh_4_)_2_	558	12	8.7	2	[11]
16	^b^ [Ni^II^(L^10^)(CH_3_CN)_2_](BPh_4_)_2_	480	11	8.4	2	[11]
17	^b^ [Ni^II^(L^11^)(CH_3_CN)_2_](BPh_4_)_2_	485	15	5.2	2	[11]
18	^b^ [Ni^II^(L^12^)(CH_3_CN)_2_](BPh_4_)_2_	365	13	5.7	2	[11]
19	^b^ [Ni^II^(L^13^)(CH_3_CN)_2_](BPh_4_)_2_	406	20	5.8	2	[11]
20	^b^ [Ni^II^(L^14^)(CH_3_CN)_2_](BPh_4_)_2_	280	34	4.7	2	[11]
21	^b^ [Ni^II^(L^15^)(CH_3_CN)](BPh_4_)_2_	487	46	10.6	2	[12]
22	^b^ [Ni^II^(L^16^)(CH_3_CN)](BPh_4_)_2_	453	62	7.3	2	[12]
23	^b^ [Ni^II^(L^17^)(CH_3_CN)](BPh_4_)_2_	424	60	7.1	2	[12]
24	^b^ [Ni^II^(L^18^)(CH_3_CN)](BPh_4_)_2_	404	41	9.9	2	[12]
25	^b^ [Ni^II^(L^19^)(CH_3_CN)](BPh_4_)_2_	272	30	9.1	2	[12]
26	^c^ [Ni^II^_2_(L^20^)_2_(μ-OH)_2_]	227	7	32	2	[13]
27	^c^ [Ni^II^_2_(L^21^)_2_(μ-OH)_2_]	210	4	53	2	[13]
28	^d^ [Ni^II^_2_(L^22^)_2_(μ-OH)_2_]	0	0	0	2	[13]
29	^d^ [Ni^II^_2_(L^23^)_2_(μ-OH)_2_]	0	0	0	2	[13]
30	^e^ [Ni^II^(L^30^)]	50	50	1	1	[17]

^a^ [Ni^II^] = 0.33 mM, [*m*-CPBA] = 0.33 mM, [cyclohexane] = 2.5 M in CH_2_Cl_2_-CH_3_CN (3:1) at 313 K under Ar. ^b^ [Ni^II^] = 0.35 mM, [*m*-CPBA] = 0.35 mM, [cyclohexane] = 2.45 M in CH_2_Cl_2_-CH_3_CN (3:1) at 313 K under N_2_. ^c^ [Ni^II^] = 1.08 mM, [*m*-CPBA] = 0.11 mM, [cyclohexane] = 5.42 mM in CH_2_Cl_2_ at 313 K under Ar. ^d^ [Ni^II^] = 1.08 μM, [*m*-CPBA] = 0.11 mM, [cyclohexane] = 5.42 mM in CH_2_Cl_2_ at 313 K under Ar. ^e^ [Ni^II^] = 0.7 mM, [*m*-CPBA] = 97 mM, [cyclohexane] = 0.62 M in CH_3_CN at 313 K. * A total TON of 300 was estimated graphically from the authors’ plot, and alcohol and ketone TONs were determined accordingly using the reported A/K ratio.

## 3. Fundamental Reactivity Studies

While much progress has been made in the catalytic hydroxylation of alkanes using mononuclear Ni^II^ catalysts, typically harsh oxidants such as *m*-CPBA were required. This prompted investigation of oxygen and its reduced forms as oxidants in the interest of developing more sustainable oxidation processes. Similar to the development of iron and copper oxidation chemistry, oxygen activation has typically been studied in nickel complexes with biologically inspired N-donor ligands such as pyridines, imidazoles, pyrazoles, etc. These families of ligands are generally known to support multiple metal oxidation states and enable rich coordination chemistry. However, Ni^II^ is generally unreactive toward dioxygen unless electron-rich ligands are employed to depress the Ni^III/II^ redox couple [20]. Of the three nickel metalloenzymes that perform oxidative chemistry, none utilize the Ni^I^ state, and there has only been one example of direct binding of dioxygen to nickel in the Ni^II^ state in nature to date [21]. As such, much of the work utilizing dioxygen as the oxidant has been carried out with Ni^I^ precursors. Through the use of redox noninnocent ligands or alternative oxidants such as peroxide or superoxide, however, interesting oxidation chemistry has been reported with nickel, even from the Ni^II^ state. In the subsequent sections, we will summarize the progress made in Ni-O_2_ chemistry toward hydrocarbon oxidation employing low-valent nickel precursors.

### 3.1. Ni-O_2_ Species

**Figure 2:**
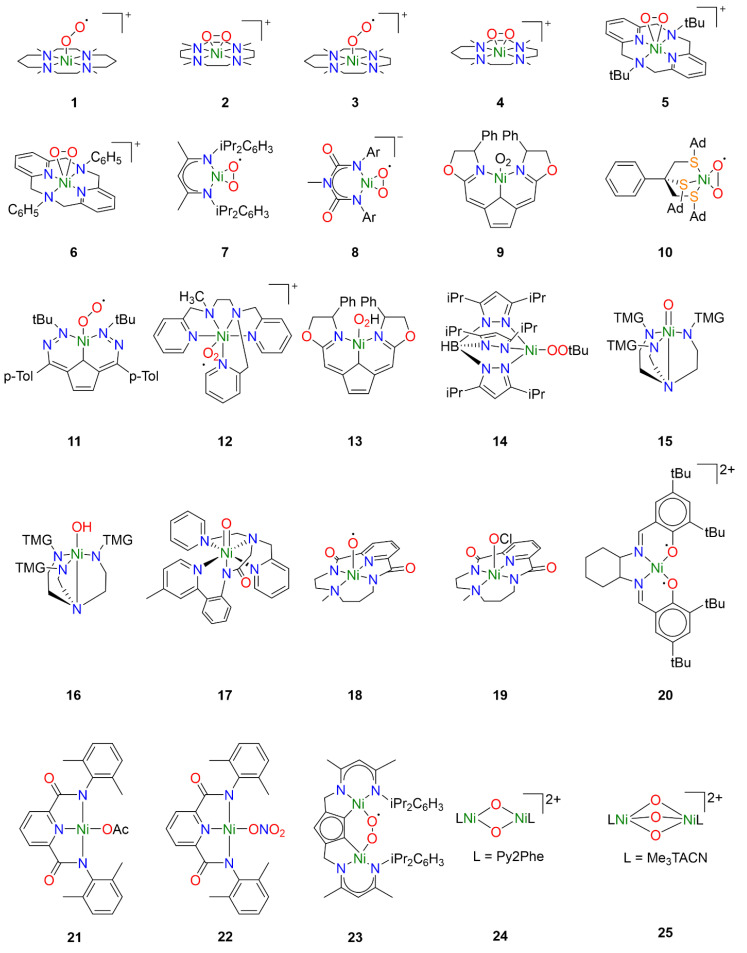
Reactive nickel-oxygen species reported in the literature.

One of the earlier investigations into Ni-O_2_ chemistry employed nickel complexes supported by the tetramethylcyclam ligand 1,4,8,11-tetramethyl-1,4,8,11tetraazacyclotetradecane (14-TMC). Treatment of [Ni^I^(14-TMC)](OTf) with excess oxygen led to the formation of the first reported end-on Ni^II^-superoxo species **1** (Figure 2) [22]. The metastable species **1** could alternatively be synthesized by treatment of the Ni^II^ precursor analogue [Ni^II^(14-TMC)]X_2_ (X = OTf^−^, ClO_4_^−^) with H_2_O_2_ in the presence of NEt_3_. Spectroscopic characterization established an *S* = ½ ground state for **1** attributed to antiferromagnetic coupling of a high-spin Ni^II^ center with the superoxide ligand radical. Following spectroscopic characterization, the reactivity of **1** was explored towards exogeneous substrates. While quantitative conversion of PPh_3_ to OPPh_3_ was observed in the presence of **1**, no reactivity towards sulfides or olefins was observed, hence its reactivity toward alkanes was not investigated.

Interestingly, by varying the size of the azacyclam ring, different Ni-O_2_ adducts could be obtained. By replacing 14-TMC with the smaller ligand 1,4,7,10-tetramethyl-1,4,7,10-tetraazacyclododecane (12-TMC), Cho et al. were able to produce the side-on formally Ni^III^-peroxo species **2** by treatment of [Ni^II^(12-TMC)(CH_3_CN)]^2+^ with H_2_O_2_ in the presence of NEt_3_ at 0 °C [23]. Complex **2** was stable at room temperature for several days, allowing for the isolation of crystals. Spectroscopic analysis supported oxidation of Ni^II^ to Ni^III^ and confirmed the *S* = ½ ground state expected in the case of the Ni^III^-peroxo species. The reactivity of **2** was investigated in both electrophilic and nucleophilic reactions. Unlike **1**, complex **2** exhibited no electrophilic reactivity and no oxidation of substrates such as PPh_3_, thioanisole, or xanthene was observed. Upon addition of 2-phenylpropionaldehyde (2-PPA), however, the corresponding deformylated product acetophenone was obtained. Subsequent Hammett analysis of the reactivity of **2** employing *para*-substituted benzaldehydes (*p*-X-Ph-CHO; X = Me, F, H, Br, Cl) afforded a positive *ρ* value of 6.1, consistent with nucleophilic character in the oxidation of aldehydes.

In a follow-up study employing the TMC ligand 1,4,7,10-tetramethyl1,4,7,10-tetraazacyclotridecane (13-TMC), both end-on Ni^II^-superoxo species and side-on Ni^III^-peroxo species could be obtained depending on which base was used [24]. When the Ni^II^(TMC) precursor was treated with H_2_O_2_ in the presence of tetramethylammonium hydroxide (TMAH), end-on Ni^II^-superoxo species **3** was obtained while side-on Ni^III^-peroxo species **4** was obtained when the same precursor was treated with H_2_O_2_ in the presence of NEt_3_. Reactivity studies confirmed electrophilic reactivity in the case of **3** and nucleophilic reactivity in the case of **4**, corroborating the previously observed trends in reactivity for Ni-O_2_ species.

Analogous side-on Ni^III^-peroxo species **5** and **6** could be obtained from Ni^II^ precursors supported by macrocyclic diazapyridinophane ligands following treatment with H_2_O_2_ in the presence of NEt_3_ [25]. Spectroscopic analysis confirmed an *S* = ½ ground state consistent with a formally Ni^III^-peroxo species. No reactivity was observed when **5** was treated with PPh_3_, thioanisole, and cyclohexadiene, indicating that **5** does not act as an electrophile. The nucleophilic reactivity of complexes **5** and **6** were investigated towards 2-PPA. In the presence of **5**, acetophenone was obtained with a yield of 71 ± 5%, while acetophenone was obtained with a yield of 90 ± 5% in the presence of **6**. Hammett analysis of the reactivity of **5** and **6** employing *para*-substituted benzaldehydes (*p*-X-Ph-CHO; X = Me, F, H, Cl, Br) afforded *ρ* values in the range of 4.3–4.4, consistent with nucleophilic character in the oxidation of aldehydes. The authors primarily attributed the higher reactivity of **6** to steric effects, as they concluded that electronic effects are likely negligible since almost identical *ρ* values were obtained from Hammett analysis of both complexes.

Similar work was also carried out with nickel complexes supported by β-diketiminate (BDI) ligands. Treatment of [(Ni^I^_2_(BDI)_2_(μ-η_3_:η_3_-C_6_H_5_Me)] with dry O_2_ in toluene afforded the side-on Ni^II^-superoxo complex **7** [26,27]. In contrast to complexes **1** and **3**, spectroscopic and computational analysis concluded that complex **7** consists of a superoxide radical bound to a low-spin diamagnetic Ni^II^ center, not unsurprising for square-planar Ni^II^. Reactivity studies concluded that **7** was competent for the oxidation of O-H and N-H bonds, though oxidation of C-H bonds of exogenous substrates was not observed. Notably, complex **7** exhibited dioxygenase-like reactivity and both oxygen atoms from a single Ni-O_2_ were incorporated into the product when **7** was exposed to para-substituted di-*tert*-butylphenols. Furthermore, the oxidation of 2,4,6-di-*tert*-butylphenol was proposed to proceed through a [NiO]^+^ intermediate formulated as a formally Ni^III^-oxo species [27]. A variation of the BDI ligand was used to stabilize the monoanionic Ni^II^-superoxo complex **8** following treatment of the Ni^II^ precursor with H_2_O_2_ in the presence of NEt_3_ [28]. While the description of the electronic structure of **8** is also best described as a *S* = ½ superoxide radical coupled to a low-spin Ni^II^ center, complex **8** exhibited nucleophilic reactivity in contrast to **7**. Whereas complex **7** could perform electrophilic hydrogen-atom transfer (HAT) reactions with weak N-H and O-H bonds, monoanionic **8** exhibits nucleophilic reactivity towards carbonyl atoms resulting in the deformylation of aldehydes, and no electrophilic reactivity was observed.

Computational studies were carried out to understand the difference in observed reactivity. In the case of **7**, the ground state was determined to be a low-spin Ni^II^ center coupled to an O_2_ ligand radical, consistent with the spectroscopic determination of the ground state. Two low-lying high-spin excited states could be identified, however, corresponding to the ferromagnetic and antiferromagnetic coupling of a high-spin Ni^II^ center coupled to the O_2_ ligand radical, respectively. The transition from low-spin to high-spin Ni^II^ is accompanied by a change in coordination geometry from square planar to tetrahedral. The excited state resulting from antiferromagnetic coupling of the Ni and O_2_ spins is characterized by large negative spin density on the O_2_ ligand as well as greater charges on the Ni- and O-atoms relative to the ground state. Such a state would easily lend itself to electrophilic reactivity, and ultimately a two-state reactivity model was proposed for **7** owing to the lower reaction barrier of the excited state compared to the ground state. In the case of **8**, a similar ground state consisting of a low-spin Ni^II^ center coupled to an O_2_ ligand radical was determined. However, the corresponding high-spin ground states are raised much higher in energy compared to complex **7** and are likely inaccessible. The O-atoms carry a larger negative charge in the ground state of **8** than in the ground state of **7**, which may explain the observed nucleophilic reactivity. It is interesting to note that antiferromagnetic coupling of the high-spin Ni^II^ center with the O_2_ ligand radical in the excited state of **5** results in a similar description of the electronic structure of complexes **1** and **3**, and in each case electrophilic reactivity is observed.

Additional tetracoordinate Ni^II^-superoxo species could also be generated from Ni^I^ precursors supported by tridentate pincer ligands. Treatment of a Ni^I^ precursor supported by a monoanionic pincer ligand with dioxygen at −40 °C afforded the Ni^II^-superoxo complex **9** [29,30]. Spectroscopic and computational studies identified two spin states of complex **9**, which were determined to be low-spin end-on and high-spin side-on Ni^II^-superoxo species, respectively, similar to complexes **7** and **8**. While ligand oxidation could be observed, complex **9** exists in a pressure-dependent equilibrium with the dinuclear 1,2-peroxo species, and ultimately it was unclear which species was responsible.

However, careful tuning of the ligand structure can favor the mononuclear Ni-O_2_ adduct over the corresponding dinuclear species. Treatment of an Ni^I^ precursor supported by the tripodal thioether ligand phenyltris((*tert*-butylthio)methyl)borate (PhTt^tBu^) with dioxygen afford the dinuclear compound [Ni^III^_2_ (PhTt^tBu^)_2_(μ-O)_2_] [31]. The resultant dinuclear compound did not react with exogenous substrates except for NO. When the ligand *t*Bu moieties were replaced by adamantane moieties, the mononuclear side-on Ni^II^-superoxo complex **10** was obtained [32]. Spectroscopic analysis determined an *S* = ½ ground state arising from antiferromagnetic coupling of a high-spin Ni^II^ center with the O_2_ ligand radical. Oxygen-atom transfer (OAT) to PPh_3_ was observed, consistent with the electrophilic reactivity observed for similar Ni^II^-superoxo species.

While typically Ni^I^ precursors are required to activate dioxygen directly, the use of redox noninnocent ligands has been reported to allow activation of dioxygen with Ni^II^ precursors. In the case of a Ni^II^ complex supported by the redox-active ligand 2,5-bis((2-*t*-butylhydrazono)(*p*-tolyl)methyl)-pyrrole (*^t^*^Bu,Tol^DHP), treatment with O_2_ led to direct formation of the Ni^II^-superoxo species **11** by electron transfer from the ligand [33]. Spectroscopic and computational analysis concluded that the *S* = ½ ground state arises from antiferromagnetic coupling of a high-spin Ni^II^ center with the O_2_ ligand radical. Oxygen-atom transfer was observed as expected when exposed to PPh_3_, and **11** was also capable of oxidizing benzyl alcohol and toluene to benzaldehyde. Interestingly, the authors conclude that because the Ni-superoxo species is formed from a comparatively mild ligand-based redox event, the resulting complex **11** is not very oxidizing.

Though less common, another synthetic pathway to access Ni^II^-superoxo species is by treatment of a Ni^II^ precursor with a salt of the superoxide anion such as KO_2_. In the case of Ni^II^ complex **12**, the addition of KO_2_ resulted in the formation of a new species exhibiting an additional charge transfer absorption tentatively attributed to the Ni-O_2_ moiety [34]. The intermediate could not be isolated and rapidly decomposed at room temperature. The formation of the Ni^II^-superoxo intermediate was inferred by the production of H_2_O_2_ in the presence of acid, which occurs in the case that superoxide is reduced by the nickel complex. While hydrocarbon oxidation studies with these compounds are still underway by one of the authors, a similar approach has been used to generate other Ni^II^-superoxo species that were capable of hydrocarbon oxidation. Complex **11** has also been obtained from addition of KO_2_ to the Ni^II^ precursor, and was capable of oxidizing substrates such as benzyl alcohol and toluene as previously discussed [33].

### 3.2. Ni-OOR Species

While Ni-OOH adducts have been considerably less explored compared to Ni-O_2_ adducts, nickel-hydroperoxo species are well known in the literature. However, the reactivity of such compounds is less well-understood. For example, the Ni^II^-OOH analogue of complex **9** has also been reported [29,30]. Treatment of Ni^II^-hydroperoxo complex **13** with PPh_3_ resulted in slow OAT to yield OPPh_3_. Ligand oxidation was also observed following thermal decomposition.

Similarly, hydroperoxo adducts of late transition metals have been investigated for their industrial and biological relevance, though reports of Ni-OOR species remain rare. The Ni^II^-alkylperoxo complex **14** was generated by the addition of t-butylhydroperoxide (TBHP) to the corresponding Ni_2_(μ-OH)_2_ precursor at 0 °C [35]. Complex **14** was relatively stable at room temperature, thermally decomposing with a half-life of approximately 2.5 h. In the presence of external substrates, both electrophilic and nucleophilic reactivity was observed. Electrophilic oxidation could be observed in the case of PPh_3_ and CO, but not towards dimethylsulfide or olefins. Hammett analysis with a series of *para*-substituted benzaldehydes (*p*-X-Ph-CHO; X = OMe, Me, H, Cl) afforded a *ρ* value of 4.3, indicating similar nucleophilic reactivity to Ni-peroxo species **5** and **6** [25].

### 3.3. Ni-O(H) Species

While terminal Ni-O or Ni-OH intermediates have been implicated in many of the catalytic studies, few have been trapped and characterized spectroscopically. Using tetradentate tripodal ligand tris[2-(N-tetramethylguanidyl)ethyl]amine (TMG_3_tren), known to support other high-valent metal-oxo species [36], Ray et al. obtained the formally Ni^III^-oxo and Ni^III^-hydroxo species **15** and **16** by treating the Ni^II^ precursor [Ni^II^(TMG_3_tren)(OTf)] with *m*-CPBA at low temperature. Spectroscopic analysis revealed two *S* = ½ species, **15** and the protonated **16**, in yields of 85% and 15%, respectively. Compounds **15** and **16** exhibited similarly reactivity, and both were competent for OAT to PPh_3_ and HAT with several substrates with C-H BDE under 80 kcal mol^−1^.

Another example of a terminal Ni-O species that could be partially characterized was obtained from Ni^II^ precursors supported by the ligand 2-(di(pyridin-2-ylmethyl)amino)-N-(2-(5-methylpyridin-2-yl)phenyl)acetamidate) (dpap) [37]. Treatment of [Ni^II^(dpap)](ClO_4_) with *m*-CPBA at low temperature afforded complex **17**, which exhibited an *S* = ½ ground state that was putatively attributed to a low-spin formally Ni^III^-oxo species. The corresponding high-spin Ni^III^-oxo and formally Ni^IV^-oxo species could not be unambiguously ruled out, however. Interestingly, epoxides were obtained when **17** was treated with olefins and Hammett analysis with a series of *para*-substituted styrenes (*p*-X-C_6_H_4_CHCH_2_; X = OMe, Me, H, Cl, CN) afford a *ρ* value of −0.75, consistent with electrophilic OAT to exogenous olefin substrates. While the exact nature of the terminal Ni-O adduct could not be unambiguously determined, the observed reactivity clearly differentiates it from the other reactive nickel-oxygen species discussed above.

Similarly, treatment of a Ni^II^ precursor supported by a macrocyclic bis(amidate) ligand resulted in the formation of complex **18**, tentatively described as a Ni^III^-oxyl species [17]. The oxidation state of the Ni center was best described as Ni^III^ on the basis of X-ray absorption spectroscopy (XAS), though results obtained from electron paramagnetic resonance (EPR) spectroscopy were more difficult to interpret. The time-resolved EPR signal corresponding to *S* = ½ Ni^III^ was uncorrelated with the spectral features of the intermediate oxidant in time-resolved absorption measurements and was ultimately assigned to a decay product. A computational investigation suggested that **18** possesses a formally Ni^IV^-oxo ground state that is better described as a Ni^III^-oxyl species. This would result in a conventionally EPR-silent species that would explain the apparent discrepancy between the XAS and EPR results. Interestingly, this could be relevant in the other studies where formally Ni^III^-oxo species are implicated. Complex **18** was reactive toward a diverse range of substrates including alkanes and olefins and could perform both OAT and HAT. Epoxidation was observed when treated with olefins, and C-H bonds with a bond dissociation energy (BDE) of up to 80 kcal mol^−1^ could be oxidized. Hammett analysis with a series of *para*-substituted methyl phenyl sulfides (*p*-X-C_6_H_4_SCH_3_; X = Me, Cl, CN) and a series of *para*-substituted styrenes (*p*-X-C_6_H_4_CHCH_2_; X = OMe, Me, H, Cl, NO_2_) both afforded a *ρ* value of −0.86, consistent with the reactivity observed for **17**.

### 3.4. Other Mononuclear Ni Species

As described above, nickel-oxygen species exhibited diverse reactivity towards exogeneous hydrocarbon substrates, though the substrate scope was a bit narrow. Substrates with weak C-H bonds were typically oxidized independent of the initial oxygen source. Treatment of the Ni^II^ precursor supported by the same macrocyclic ligand as **18** with NaOCl in the presence of AcOH at low temperature resulted in formation of species **19** [18]. Spectroscopic and computational investigations concluded that **19** was as a formally Ni^IV^-OCl species, though perhaps better formulated as a physically Ni^III^ species with significant ligand radical character. Complex **19** could perform both OAT and HAT, and notably could oxidize exogeneous hydrocarbon substrates with strong C-H bonds such as cyclohexane.

Similar ligand-centered radicals could be observed when a Ni^II^ precursor supported by the Jacobsen salen ligand *N*,*N*′-bis(3,5-di-*tert*-butyl-salicylidene)-1,2-cyclohexane-(1*R*,2*R*)-diamine was treated with *m*-CPBA at low temperature, affording complex **20** [38]. Spectroscopic measurements suggested that **20** was best described as a Ni^III^-bisphenoxyl diradical species, corresponding to three oxidizing equivalents per compound as confirmed by ferrocenium titrations. The three unpaired electrons can antiferromagnetically couple in two ways to give two *S* = ½ species, one giving a metal-centered EPR signal and the other a ligand-centered EPR signal, and ultimately both species were observed. The ferromagnetically coupled species was not observed. Complex **20** was reactive towards substrates such as xanthene, 2,4,6-tri-*tert*-butylphenol, thioanisole, and phosphine. It was unclear whether the observed reactivity proceeds through only one of the spin isomers or both.

It is substantiated that obtaining high-valent metal intermediate might be facilitated by metal complexes supported by anionic ligands [39,40]. To this end, Ni^II^-carboxylate/nitrate complexes were investigated toward hydrocarbon oxidation. These Ni-complexes were oxidized by magic blue or cerium ammonium nitrates to obtain Ni^III^-OAc/Ni^III^-ONO_2_ species **21** and **22** that showed similar oxidizing capabilities as observed previously. Oxidation of toluene and substrates with weaker C-H could be observed.

### 3.5. Dinuclear Ni Species

While most of the literature has focused on mononuclear nickel-oxygen species, reports of dinuclear nickel-oxygen species are known. For example, treatment of a dinuclear Ni^II^ precursor supported by a modified BDI ligand with excess dioxygen at room temperature afford the μ-1,2-superoxo dinickel(II) species **23** [41]. Complex **23** was able to oxidize the O-H group of TEMPO-H at room temperature, but ultimately was not very reactive towards H-atom abstraction. Similar BDI ligands have also been reported to lead to the formation of dinuclear and polymeric organoperoxide intermediates [42].

Reports of dinuclear oxidants derived from H_2_O_2_ are more rare in the literature, though bis(μ-oxo)dinickel(III) species have been generated from the addition of H_2_O_2_ to the corresponding bis(μ-hydroxo)dinickel(II) species. This is well documented in a series of dinuclear nickel complexes supported by TPA derivatives [43,44,45,46]. In many cases, only ligand hydroxylation was reported. However, 2,4-di-*tert*-butylphenol, 2,6-di-*tert*-butylphenol, and 1,4-cyclohexadiene were oxidized to the corresponding bisphenol derivative and benzene in the presence of **24** following treatment with H_2_O_2_ [45].

Formally, dinuclear Ni^IV^-oxido species have also been reported, obtained by treatment of a dinuclear Ni^II^ precursor to afford the [(L)_2_Ni^IV^_2_(μ-O)_3_]^2+^ species **25** [47]. Oxidative reactivity toward substrates such as xanthene, fluorene, and 9,10-dihydroanthracene was reported, though it was noted that these substrates may also be oxidized in the presence of NaOCl.

## 4. Oxidative Chemistry of Nickel-Containing Metalloenzymes

Nickel is employed by biological systems to perform a wide range of chemistry, from redox-active reductive and oxidative processes to redox-neutral isomerization and hydrolysis reactions. Of the ten nickel enzymes currently known, three have been reported to catalyze oxidative transformations: superoxide dismutase, acireductone dioxygenase, and quercetin dioxygenase [48,49]. Each of these has served as inspiration for synthetic model studies pushing the boundaries of the oxidative chemistry of nickel [33,50,51,52,53,54,55,56]. Here, we have chosen to highlight those aspects of these three enzymes that illustrate the diversity of nickel coordination chemistry in nature and the subtle effects that drive such a wide range of oxidative transformations. We anticipate that the study of these systems will continue to inform synthetic investigations in the development of more sustainable oxidative chemistry.

**Figure 3:**
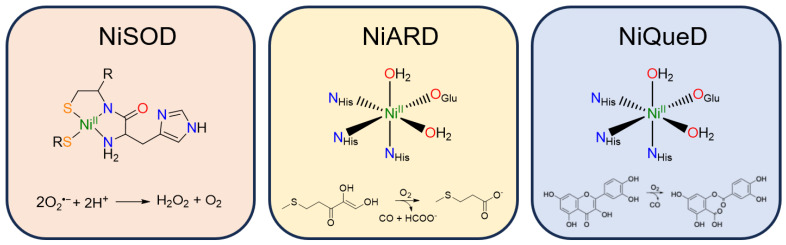
Active site structure and reaction catalyzed by the three currently known metalloenzymes that perform oxidative chemistry: nickel superoxide dismutase, nickel acireductone dioxygenase, and nickel quercetin dioxygenase.

Nickel superoxide dismutase (NiSOD) catalyzes the disproportionation of superoxide to peroxide and dioxygen (Figure 3) [57]. The Ni^II^ resting state is coordinated by a backbone amide from Cys^2^, the N-terminus primary amine, and two thiolate ligands derived from Cys^2^ and Cys^6^ [58]. Interestingly, the nickel center in NiSOD converts between a square planar N_2_S_2_ coordination environment in the Ni^II^ state and a square pyramidal N_3_S_2_ coordination environment in the Ni^III^ state in which axial binding of the His^1^ imidazole to the nickel center is induced following oxidation [57,59,60]. While most SODs employ manganese, iron, or copper for redox catalysis, the Ni^III/II^ redox couple in NiSOD is lowered by more than 1 V relative to typical Ni^III/II^ redox couples in aqueous solution, generally attributed to the unique coordination environment and active site hydrogen bonding network [61].

Superoxide disproportionation catalyzed by NiSOD proceeds through a ‘ping-pong’ electron transfer mechanism [53]. Superoxide initially binds the Ni^II^ center, generating a Ni^II^-peroxo species that undergoes electron and proton transfer to generate H_2_O_2_, leaving the nickel center in the Ni^III^ state. There are several nearby residues that have been suggested as feasible proton sources. Following oxidation, the nickel center is additionally coordinated by His^1^ and the resulting square-pyramidal Ni^III^ center binds an additional superoxide. Electron transfer from superoxide to Ni^III^ produces dioxygen and regenerates the Ni^II^ resting state, completing the catalytic cycle.

Nickel acireductone dioxygenase (NiARD) catalyzes the off-pathway production of carbon monoxide and 3-methylpropionic acid (MTP) from acireductone (Figure 3) in the penultimate step of the methionine salvage pathway [62,63,64]. The active site contains an octahedral, high spin Ni^II^ center coordinated by three histidines and glutamic acid in a 3-His-1-carboxylate motif and two water molecules (Figure 3) [58,65,66,67,68]. Several mechanisms have been proposed, and while the mechanism remains an open question, it is generally agreed upon that the role of Ni^II^ in NiARD is that of a Lewis acid [64,68,69]. Upon binding acireductone in its dianionic form, the bound acireductone proceeds to react with O_2_, forming a cyclic peroxide as an intermediate which subsequently decomposes to yield carbon monoxide, formic acid, and MTP [62,63]. Direct interactions between the Ni^II^ center of NiARD and O_2_ have not been observed. Model studies exhibiting similar chemistry have suggested that Ni^II^ is important in stabilizing the acireductone dianion and producing a biologically appropriate rate of reaction with O_2_ [50,51]. As acireductone anions are known to react with O_2_ even in the absence of enzyme [55], the formation of a Ni^II^-bound substrate is likely required to control the rate of the reaction.

**Figure 4:**
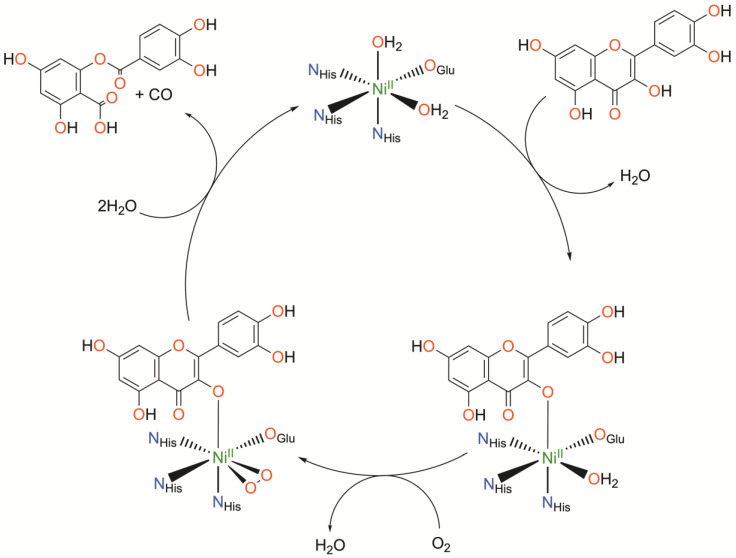
Catalytic cycle of nickel quercetin dioxygenase as determined crystallographically. Charges omitted for clarity.

Nickel quercetin dioxygenase (NiQueD) catalyzes the oxidative ring-cleaving of quercetin, releasing carbon monoxide (Figure 3) [20]. Like NiARD, the active site of NiQueD contains an octahedral, high spin Ni^II^ center coordinated by a similar 3-His-1-carboxlyate motif and two water molecules (Figure 3). However, the role of the Ni^II^ center is less clear. Unlike NiARD, NiQueD has been shown to bind O_2_ directly [21], while reactivity towards O_2_ is uncommon for the Ni^II^ oxidation state [70,71]. This was demonstrated by Jeoung et al. using a crystallographic cryotrapping approach in which binding of quercetin and simultaneous binding of quercetin and O_2_ could be observed [21]. From this, the following catalytic cycle was proposed (Figure 4).

Initial binding of quercetin replaces the axially coordinated water and results in a lengthening of the Ni-O bond distance of the equatorially coordinated water. The coordinating carboxylate rotates approximately 90°, bringing the second oxygen atom (Oε2) of E-76 within hydrogen bonding distance with quercetin such that quercetin is subsequently deprotonated. Upon exposure to O_2_, dioxygen binds the Ni^II^ center in a side-on fashion, replacing the equatorially coordinated water. This is followed by attack of the activated O_2_ on quercetin, ultimately releasing CO and the depside resulting from quercetin oxidation. The authors conclude that Ni^II^ serves to both activate and align both substrates for the reaction. Binding of quercetin to the Ni^II^ center stabilizes the deprotonated, anionic state of quercetin while simultaneously priming the Ni^II^ center for binding of O_2_. The authors suspect the possible formation of a Ni^II^-superoxo intermediate based on the crystallographically determined O-O bond length.

A combined quantum mechanics/molecular mechanics (QM/MM) investigation starting from the crystal structure of the O_2_-NiQueD-quercetin complex employing DFT for the active site, however, concluded that O_2_ binds in an end-on manner following QM/MM optimization, and all attempts to obtain side-on binding of O_2_ converged to the monodentate end-on binding mode. From a close analysis of the spin density of the O_2_-NiQueD-quercetin complex in the triplet and quintet states, it was determined that formation of the triplet state is consistent with transfer of an electron from quercetin to O_2_ and formation of a Ni^II^-superoxo species following binding of O_2_ [72]. This is accompanied by a modulation of the Ni spin density, and the authors conclude that the electron is transferred from quercetin to O_2_ via the nickel ion with no net change in the formal oxidation state of the nickel ion [72]. Similar chemistry has been observed in NiQueD model complexes employing redox-active ligands, demonstrating the feasibility of the mechanism proposed for NiQueD and the viability of accessing Ni^II^-superoxo species from Ni^II^ precursors by exploiting ligand noninnocence [33]. Interestingly, while quercetin oxidation has been reported for one NiQueD model complex, the mechanism remains unclear [56].

## 5. Conclusions

In summary, the past two decades have seen considerable development in nickel-mediated oxidative chemistry. Many research groups around the world reported alkane oxidation with high TON, product selectivity, and regioselectivity using mononuclear nickel precursors. Much of what we know about the coordination chemistry of nickel and high-valent nickel intermediates is a direct result of this body of work. In the case of mononuclear nickel complexes, varying the ligand structure in a systematic manner proved to be an effective approach and trends across different ligand families could be established to improve catalytic performance in some cases. In almost all the catalytic studies presented, an [NiO]^+^ species was implicated, though in many cases a detailed description of the electronic structure was unclear. We imagine that further spectroscopic and computational investigation of these intermediates will be necessary to understand and optimize their reactivity.

Reactivity studies of metalloenzyme-inspired model complexes have played a crucial role in understanding the oxidative chemistry of metals such as iron and copper. With the recent discovery of nickel quercetin dioxygenase, the same kind of synergistic approach between biochemical and bioinspired synthetic studies can already be observed and has already led to interesting insights into nickel-dioxygen chemistry. We expect that the knowledge acquired in these experiments will lead to further developments in both the fundamental coordination chemistry and metallobiochemistry of nickel.

The current literature raises a few interesting questions, particularly in the case of formally Ni^III^-O and Ni^IV^-O species. There are few reports of these terminal Ni-O adducts that have been characterized in the literature, and much is still unknown. While many of the reported studies have relied on density functional theory to understand the electronic structure of such terminal Ni-O species, these systems are known to often require higher levels of theory to accurately describe them. Based on a close reading of the available literature, it seems that wavefunction-based quantum chemical methods may be necessary to advance our understanding of these species.

Though most of the reviewed work has focused on mononuclear nickel-oxygen species, revisiting the study of dinuclear nickel-oxygen intermediates with the knowledge gained over the last few years might prove particularly fruitful. Finally, while many of the reported catalytic studies in the literature have employed mCPBA as the oxidant, much work has been done in fundamental reactivity studies with dioxygen and reduced forms of dioxygen as the oxidant, and further exploration with these and alternative oxidants will be crucial for developing more sustainable oxidative chemistry. Overall, the field remains relatively young, and we look forward to the new discoveries that will be made.
